# Cancer Center Shared Resources: A Nexus for Discovery, Innovation, and Impact

**DOI:** 10.7171/001c.166361

**Published:** 2026-09-01

**Authors:** Sheenah Mische, Jay W. Fox, Nicholas P. Ambulos

**Affiliations:** 1 Division of Advanced Research Technologies NYU Langone’s Laura and Isaac Perlmutter Cancer Center; 2 Administration University of Virginia Cancer Center; 3 Shared Resource Administration University of Maryland Marlene and Stewart Greenebaum Comprehensive Cancer Center

**Keywords:** NCI designated Cancer Centers, Shared Research Resources, innovation, National Association of Cancer Center Shared Resources, advanced technologies

## Abstract

Shared Research Resources (SRRs) are indispensable to modern cancer research, powering discovery through advanced technologies, specialized expertise, and strategic scientific partnership. At NCI-designated Cancer Centers, their role has expanded from core service platforms to drivers of innovation, rigor, and translational impact. In 2024, this evolution led to the creation of the National Alliance of Cancer Center Shared Resources (NACCSR), a national collaboratory uniting Shared Resource leaders to advance best practices, strengthen partnerships, and shape the future of the field. The articles in this special issue highlight key priorities including metrics, management, data and metadata stewardship, cross-institutional collaboration, and emerging technologies. Together, they position SRRs as essential architects of the next era of cancer research—one defined by innovation, reproducibility, FAIR data, and collective impact. NACCSR stands poised to help lead that transformation.

Shared research resources (SRRs) are essential to efficient and effective support for high-impact research. In today’s competitive biomedical research environment, successful discovery depends on access to state-of-the-art technologies and specialized expertise, while translating these discoveries into patient and population benefits relies equally on the advanced capabilities SRRs provide. Nowhere is this more evident than in modern academic cancer centers.

Through the Cancer Centers Program, the National Cancer Institute (NCI) has long viewed SRRs as a cornerstone of scientific excellence within NCI-designated cancer centers. Historically, Cancer Center Support Grants (CCSGs) were often referred to as “the core grant” because of the substantial investment directed toward SRRs. Over time, this emphasis has expanded beyond the resources themselves to include strategic oversight and management. Today, NCI emphasizes strategic planning, evaluation, and governance of SRRs — collectively termed shared resource management — to ensure resource portfolios remain aligned with each cancer center’s evolving scientific priorities and strategic goals. In 2024, the guest editors of this special issue of the *Journal of Biomolecular Techniques* convened leaders to establish a national collaboratory through which Shared Resource leaders could share ideas, experiences, best practices, and outcomes with colleagues across NCI-designated cancer centers. The resulting National Alliance of Cancer Center Shared Resources (NACCSR) serves not only to connect and leverage the collective expertise of shared resource leaders, but they also serve to foster greater alignment and collaboration with national organizations and initiatives, including the NCI Office of Cancer Centers, the Association of Biomolecular Resource Facilities, the Cancer Informatics Society, the Association of Cancer Center Administrators, and the American Association for Cancer Research.

The articles in this special issue span the breadth and diversity of perspectives that NACCSR members bring to advancing the value of SRRs within cancer centers and through partnerships with other national organizations. The article by Chipuk and colleagues highlights the work of the NACCSR Metrics Working Group and reports on a first-of-its-kind survey of the key performance metrics used across cancer centers to assess the effectiveness and impact of shared resources. Additional NACCSR member contributions address the complexities of shared resource management within the NCI-designated cancer center environment, innovations in data management and sharing to strengthen data and metadata production in SRRs, strategies for resource sharing and collaboration across institutions, and the broader impact of shared resources beyond their traditional role in supporting programmatic research. And, as technology serves as the primary engine for modern research, we present two articles on future technology for shared resources.

Over the past several decades, SRRs have evolved from service providers into indispensable partners in discovery and drivers of high-impact science. That evolution is ongoing. As cancer centers navigate a rapidly shifting scientific and technological landscape, shared resource leaders will play a central role in shaping the future of NCI-designated cancer centers. As major producers and stewards of research data, SRRs are essential to ensuring data quality, provenance, and metadata capture, thereby enabling data to be rigorous, reproducible, and increasingly FAIR (findable, accessible, interoperable, and reusable). Advances, such as artificial intelligence, will create new opportunities for SRRs to align with institutional priorities, accelerate innovation, and broaden their contributions across therapeutic development, education/training, data science, and community engagement. As the next era of cancer research unfolds, one reality is already clear: NACCSR members will be instrumental in defining and driving that future.

Sheenah, Jay, & Nick

## Financial Support/Conflict of Interest

The authors declare no conflicts of interest or competing financial interests.

